# 2024 FDA TIDES (Peptides and Oligonucleotides) Harvest

**DOI:** 10.3390/ph18030291

**Published:** 2025-02-20

**Authors:** Othman Al Musaimi, Danah AlShaer, Beatriz G. de la Torre, Fernando Albericio

**Affiliations:** 1 School of Pharmacy, Newcastle University, Newcastle upon Tyne NE1 7RU, UK; 2 Department of Chemical Engineering, Imperial College London, London SW7 2AZ, UK; danah.shaer@gmail.com; 3 School of Laboratory Medicine and Medical Sciences, College of Health Sciences, University of KwaZulu-Natal, Durban 4001, South Africa; garciadelatorreb@ukzn.ac.za; 4 School of Chemistry and Physics, University of KwaZulu-Natal, Durban 4001, South Africa; 5 Department of Organic Chemistry, University of Barcelona, 08028 Barcelona, Spain

**Keywords:** FDA approvals, drugs, peptides, oligonucleotides, imetelstat, palopegteriparatide, levacetylleucine, pegulicianine

## Abstract

In 2024, the FDA approved fifty novel drugs, including four peptides and oligonucleotides (TIDEs) (two pepTIDEs and two oligonucleoTIDEs), highlighting their increasing importance as effective alternatives to traditional drug classes. TIDEs provide essential therapies for complex diseases, such as genetic disorders, rather than merely addressing symptoms. In addition to oligonucleotide therapeutics for various genetic conditions, peptides became the first approved treatment for Rett Syndrome in 2023 and were also used to treat Niemann–Pick disease type C (NPC) in 2024. Interestingly, among the strategies employed in recent approvals to enhance stability and/or delivery, the prodrug approach, exemplified by palopegteriparatide and pegulicianine, is emerging as a more targeted and precise therapeutic strategy. Additionally, the Enhanced Stabilization Chemistry (ESC)-GalNAc platform has been expanded for hepatic delivery of a new oligonucleotide drug, olezarsen. Furthermore, novel modifications to the ribose moiety in oligonucleotides, such as the 3′-amino substitution in imetelstat, enhance their stability. This review examines the TIDES approved in 2024 based on their chemical structure, medical targets, modes of action, administration routes, and common adverse effects. In addition, it highlights how the prodrug strategy has improved targeting efficiency and extended the half-lives of the active drugs.

## 1. Introduction

**Figure 1:**
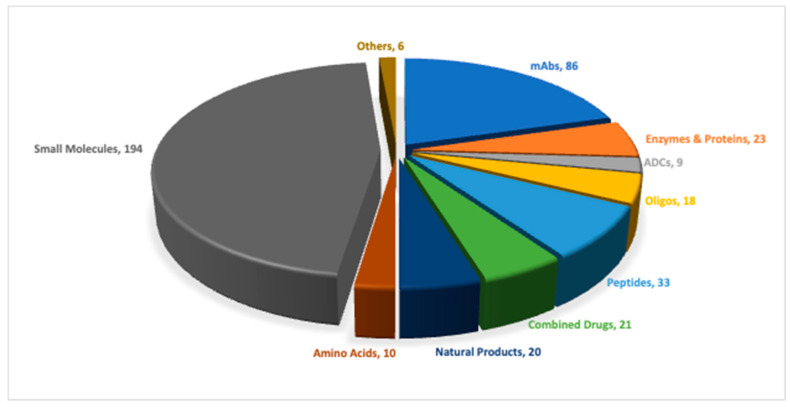
A total of 420 new drugs were approved by the Food and Drug Administration (FDA) between 2016 and 2024. ADCs, antibody–drug conjugates; mAbs, monoclonal antibodies; Oligos, oligonucleotides.

As of this year, the Food and Drug Administration (FDA) approved a total of fifty new molecular entities (NMEs), comprising thirty-four new chemical entities (NCEs) (including two oligonucleotides, two peptides, and thirty small molecules) and sixteen biologics (monoclonal antibodies, antibody–drug conjugates, enzymes, and proteins), adding up to a total of four hundred and twenty NME approvals in the past 9 years (Figure 1) [1].

Oligonucleotide therapeutics, owing to their sequence specificity, enable the selective modulation of RNA targets that were previously considered undruggable by small molecules, which require defined binding pockets on proteins, and biologics, which are largely restricted to extracellular targets. This capability positions them as powerful precision medicines, particularly for genetic and neurodegenerative disorders [2,3].

The first FDA-approved oligonucleotide therapeutic was fomivirsen (Vitravene™), an antisense oligonucleotide (ASO) approved in 1998 for the treatment of cytomegalovirus (CMV) retinitis in immunocompromised patients, particularly those with AIDS. Since then, 21 oligonucleotide therapeutics have received FDA approval as of 2024 [4]. These therapeutics address a range of disorders via diverse mechanisms of action, encompassing eleven antisense oligonucleotides (ASOs), three aptamers, six small interfering RNAs (siRNAs) [4], and one compound, defibrotide (Defitelio™), whose precise mechanism of action remains unclear [5].

Despite challenges related to cellular uptake and biodistribution, advancements in delivery technologies, such as lipid nanoparticles and GalNAc conjugates, have significantly improved the stability and tissue specificity of oligonucleotide therapeutics. Their growing clinical success is exemplified by patisiran (Onpattro™) [6], the first FDA-approved siRNA therapeutic and the first to utilize lipid nanoparticle delivery, as well as givosiran (Givlaari™) [7], the second approved siRNA therapy and the first to employ the Enhanced Stabilization Chemistry (ESC)–GalNAc conjugation for targeted liver delivery. These and other examples highlight the potential of oligonucleotide therapeutics to complement or expand the treatment landscape beyond traditional drug modalities.

In 2024, two oligonucleotides, imetelstat and olezarsen, received FDA approval, highlighting significant progress in the field. Imetelstat, a telomerase inhibitor, introduces a novel amino-substituent at the 3′ position [8], a modification designed to enhance the oligonucleotide stability [9], while olezarsen, targeting familial chylomicronemia syndrome (FCS), employs a GalNAc-containing ligand for precise liver-targeted delivery—advancing innovations in oligonucleotide therapeutics.

Peptides, situated between small molecules and biologics, offer high specificity, lower immunogenicity, and cost-effective production. With around 120 peptide drugs on the market and many in development, they play vital roles in diagnosis, drug discovery, and immunology. Peptides act as hormones, neurotransmitters, and signaling molecules, combining affinity, specificity, and low immunogenicity, making them invaluable in medicine [1].

Peptide science began in 1882 with Curtius’s synthesis of benzoylglycylglycine, followed by Fischer’s synthesis of the first free dipeptide and the term “peptide” in 1901 [10,11]. Peptides gained therapeutic significance with Banting’s isolation of insulin in 1921 and have since expanded beyond natural sources to include novel modified structures [12].

Peptide therapeutics are entering a new era, driven by technological advancements, scientific insights, and clinical successes. Since their inception in the 1920s, peptides have evolved into a versatile drug class capable of addressing complex diseases [13]. Their origins have expanded beyond natural endogenous forms, integrating seamlessly into medicinal chemistry to produce innovative and modified structures [14]. Advancements in synthetic methods, particularly solid-phase peptide synthesis (SPPS), have streamlined production and significantly expanded the pipeline of peptides progressing through clinical development [15]. However, their inherently short half-lives remain a challenge. To address this, researchers are exploring various stabilization strategies, including cyclization, lipidation, and pegylation, to enhance their therapeutic potential [16]. Table 1 provides a summary of the TIDES approved in 2024.

In this review, we aim to examine the FDA approvals of TIDES in 2024, with a focus on their indications, therapeutic targets, mechanisms of action, administration routes, and potential side effects.

## 2. Oligonucleotides

This year concluded with a harvest of two new approved oligonucleotides, marking significant progress in the field. Imetelstat is particularly noteworthy for its novel chemical structure, featuring an amino-substituent at the 3′ position—an unprecedented modification among FDA-approved oligonucleotides [8,22]. The second approval this year, olezarsen, represents a milestone as the first ASO oligonucleotide specifically targeting FCS. Its design incorporates a GalNAc-containing ligand that binds the asialoglycoprotein receptor (ASGPR) in hepatocytes, facilitating targeted delivery [23].

This liver-targeting strategy has recently gained traction in oligonucleotide therapeutics. It is employed either through dendrimeric ligands conjugated to the strand’s terminus—utilizing the ESC-GalNAc platform, as demonstrated in several recent approvals—or through substituents directly attached to the ribose unit of specific monomers within the sequence. For instance, nedosiran leverages the GalXC™ platform, in which GalNAc is conjugated directly to the RNA strand, further exemplifying the innovation in this domain [24].

The GalNAc approach presents distinct advantages over nanoparticle-based delivery methods, such as enhanced potency, greater cost-efficiency in production, and a decrease in inflammatory side effects [25].

### 2.1. Imetelstat (RyteloO™)

**Figure 2:**
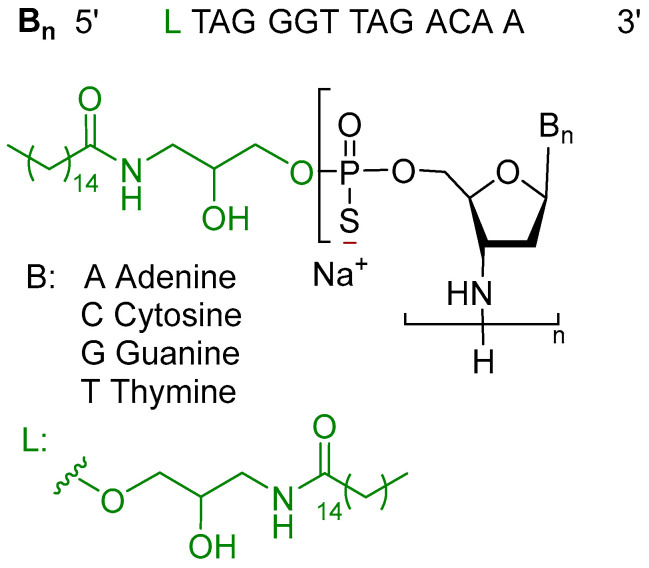
Chemical structure of imetelstat (Rytelo™).

Imetelstat is an antisense oligonucleotide, comprising the sodium salt of the first-of-its-kind 13-mer single-stranded DNA. Each monomer within the strand features a 3′-amino group substituent and is connected through phosphorothioate linkages. The 5′-terminus is conjugated to a palmitoyl lipid chain via an aminopropane diol spacer (Figure 2) [22].

Imetelstat is indicated for the treatment of adult patients with low- to intermediate-1 risk myelodysplastic syndromes (MDSs) and transfusion-dependent anemia for whom erythropoiesis-stimulating agents (ESAs) are unsuitable [22]. MDS is a group of rare hematologic disorders characterized by ineffective hematopoiesis (blood cell production), resulting in the production of excessive immature blood cells, known as blast cells, and insufficient healthy blood cells. This leads to chronic anemia, often requiring regular blood transfusions to maintain hemoglobin levels and manage symptoms such as fatigue. Although ESAs are commonly used to enhance red blood cell production, some patients may not respond adequately to these treatments, relying solely on transfusions and necessitating alternative therapeutic options [8].

Telomerase, an enzyme responsible for proliferative immortality, is upregulated in nearly all cancer cells [8]. Imetelstat targets the RNA template of telomerase, binding through a complementary sequence to inhibit its function in adding telomeric repeats to chromosome ends. This inhibition disrupts the enzyme’s ability to maintain chromosomal integrity, ultimately halting cell division. Through this mechanism, imetelstat reduces disease progression and supports disease stabilization [26,27]. This mechanism was confirmed through the pharmacodynamic profile observed in both in vitro and in vivo studies [8,28].

Imetelstat exhibits a consistent pharmacokinetic profile, reaching a mean plasma Cmax of 18.3 μM without accumulation between doses. It is highly bound to plasma proteins (94%) and has a broad volume of distribution (Vd ~14.1 L), undergoing metabolism by nucleases. With a half-life of 4.9 h, it necessitates dosing every four weeks. The drug is eliminated through urine, with no unchanged drug detected, and it inhibits the organic anion transporting polypeptide 1B1 (OATP1B1) and organic anion transporting polypeptide 1B3 (OATP1B3), potentially influencing interactions with other medications [8,28].

In phase II studies, imetelstat (Group 1: 8.9 mg/kg and Group 2: 4.4 mg/kg) led to better symptom response and overall survival in myelofibrosis patients compared to placebo. At week 24, 32.2% of patients in Group 1 showed symptom improvement, compared to only 6.3% in Group 2. Spleen response was observed in Group 1 (10.2%), but not in Group 2. The median overall survival was 29.9 months for Group 1 and 19.9 months for Group 2, after a median follow-up of 27.4 months. However, the higher dose in Group 1 was associated with a higher occurrence of hematologic adverse events [28]. In a phase II/III study for low-risk myelodysplastic syndrome (LR-MDS), imetelstat (7.1 mg/kg every 4 weeks) significantly improved red blood cell transfusion independence compared to placebo, with a notable improvement in both short-term and long-term transfusion independence. Despite frequent grade 3/4 cytopenias, the safety profile was manageable with dose adjustments, and severe infections or bleeding were rare [28].

Rytelo^TM^ is administered via intravenous infusion. Its potential adverse effects include thrombocytopenia, leukopenia, neutropenia, elevated aspartate aminotransferase (AST), increased alkaline phosphatase, elevated alanine aminotransferase (ALT), fatigue, prolonged partial thromboplastin time, arthralgia/myalgia, COVID-19 infections, and headache [22].

Imetelstat, previously known as GRN163, was first developed as a universal anticancer agent at the University of Nebraska Medical Center under contract with Lynx Therapeutics [27], was developed by Geron corporation, and approved by FDA on 6 June 2024 [17].

### 2.2. Olezarsen (Tryngolza™)

**Figure 3:**
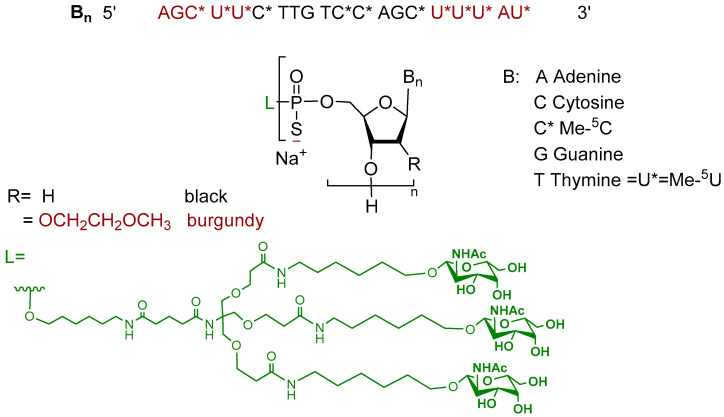
Chemical structure of olezarsen (Tryngolza™).

Olezarsen is an antisense oligonucleotide composed of the sodium salt of a 20-mer DNA-RNA single strand. The strand features a hybrid design, with half of the monomers containing 2′-deoxyribose moieties and the other half comprising five units at each terminus, incorporating 2′-methoxyethoxy-substituted ribose units. These units are connected via phosphorothioate linkages. The sequence contains five 5-methylcytosine nucleobases and six 5-methyluracil nucleobases (analogous to the thymine nucleobase but present in an RNA monomer) denoted as C* and U*, respectively (Figure 3). The oligonucleotide is conjugated at its 5′ terminus to a hepatocyte-targeting dendrimer-ligand [29]. Olezarsen represents the sixth oligonucleotide therapeutic utilizing the ESC-NGalNAc platform [4,24].

Olezarsen is indicated as an adjunct to diet to reduce triglycerides in adults with FCS [29]. FCS is a rare genetic disorder characterized by an impaired capacity to catabolize chylomicrons, which are lipoprotein particles responsible for transporting dietary triglycerides and cholesterol in the bloodstream. This impairment results in the accumulation of chylomicrons leading to severe hypertriglyceridemia, with triglyceride levels often exceeding 1000 mg/dL [25,30].

FCS is caused by mutations in the lipoprotein lipase (LPL) gene or other genes encoding proteins essential for LPL function, resulting in LPL deficiency or dysfunction. LPL is a critical enzyme for the hydrolysis of triglycerides contained within chylomicrons. Additionally, apolipoprotein C-III (APOC3) is known to inhibit LPL activity and contribute to elevated plasma triglycerides through both LPL-dependent and LPL-independent mechanisms. Inhibiting APOC3 has been shown to effectively reduce plasma triglyceride levels [25,30].

Olezarsen, as an antisense oligonucleotide, targets APOC3 mRNA, promoting its degradation via RNase H1, an endogenous ribonuclease present in mammalian cells. This targeted approach reduces APOC3 protein levels, thereby enhancing triglyceride clearance and improving plasma triglyceride concentrations [29].

Olezarsen showed peak plasma concentrations around 3–4 h after a single dose, with detectable levels for up to 150 days. The drug has a long terminal half-life (197–659 h) and exhibits extensive tissue distribution (7050–12,800 L). Renal elimination is minimal, and after multiple doses, the pharmacokinetic profile remained stable with minimal accumulation. The volume of distribution and renal clearance remained consistent, indicating negligible renal excretion [31].

In the clinical trials, olezarsen at 50 mg and 80 mg significantly reduced triglyceride levels by 49.3 and 53.1 percentage points, respectively, compared to placebo (*p* < 0.001). Both doses also lowered APOC3, apolipoprotein B, and non-HDL cholesterol, with no significant impact on LDL cholesterol. Adverse events were comparable across groups, with rare hepatic, renal, or platelet abnormalities [32].

In another study, where 71% of patients had a history of acute pancreatitis, participants were divided into three groups: 80 mg olezarsen, 50 mg olezarsen, and placebo. The 80 mg dose of olezarsen resulted in a significant 43.5 percentage-point reduction in triglycerides compared to placebo, while the 50 mg dose showed a smaller, non-significant reduction. Both doses significantly lowered apolipoprotein C-III levels. Acute pancreatitis episodes were much lower in the olezarsen groups (4.3%) compared to placebo (48%), with a rate ratio of 0.12 (*p* < 0.001). Moderate adverse events were reported in 18% of patients receiving 80 mg olezarsen [33].

Olezarsen is administered subcutaneously once per month. Its most frequently reported adverse effects include injection site reactions, reduced platelet counts, and arthralgia [29].

Olezarsen’s oligonucleotide strand was developed by Ionis Pharmaceuticals as volanesorsen [23,25]. The drug volanesorsen was approved for medical use in the European Union in May 2019 but was not approved by the US FDA due to safety concerns [34]. Volanesorsen treatment led to thrombocytopenia in approximately 45% of patients, with 6% experiencing severe platelet reductions below 25,000 per µL, though platelet counts recovered after discontinuation of the drug [35]. However, when the strand was further conjugated to a hepatocyte-targeting ligand and optimized into olezarsen, it received FDA approval on 19 December 2024 [18].

## 3. Peptides

Among the 2024 approvals, the prodrug strategy stands out as a more targeted therapy, offering drug protection while reducing side effects and off-targeting. For instance, a branched methoxypolyethylene glycol has been integrated into palopegteriparatide to shield its active component from receptor interactions, thereby extending its half-life in circulation [36]. Another example is pegulicianine, which remains inactive until cleaved by enzymes abundant around tumors and tumor-associated cells, resulting in the generation of fluorescent metabolites [37].

### 3.1. Palopegteriparatide (Yorvipath^TM^)

**Figure 4:**
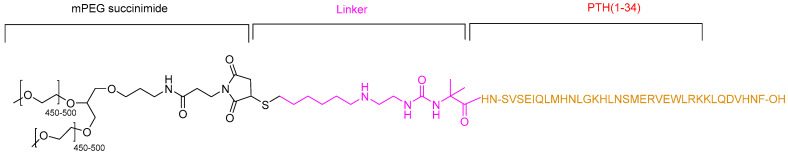
Chemical structure of palopegteriparatide (Yorvipath^TM^).

Palopegteriparatide, a prodrug also known as TransCon parathyroid hormone (PTH), is a parathyroid hormone analog (PTH(1-34)). It consists of PTH attached to a linker that binds to a carrier molecule, a branched methoxypolyethylene glycol, shielding it from receptor binding and extending its half-life in circulation (Figure 4) [36]. It is used to treat hypoparathyroidism in adults [38]. Conventional hypoparathyroidism therapy with active vitamin D and calcium addresses hypocalcemia but fails to restore normal PTH physiology [36]. This approach often results in calcium level fluctuations, hypercalciuria, renal impairment, and reduced quality of life [36,39]. PTH replacement therapy offers an ideal physiologic treatment for this condition.

At physiological pH and skin temperature, PTH(1-34) is released, mimicking endogenous secretion, while the carrier is excreted by the kidney [13,39]. It mobilizes minerals from bone, enhances renal calcium reabsorption, increases phosphate excretion, and boosts vitamin D synthesis for calcium absorption, acting via PTH1R on osteoblasts, osteocytes, and renal cells [38].

Palopegteriparatide has demonstrated improved renal function in chronic hypoparathyroidism patients [40], with significant enhancement in estimated glomerular filtration rate (eGFR) observed at week 52, alongside the maintenance and normalization of serum and urine biochemistries [40]. Palopegteriparatide significantly improved Hypoparathyroidism Patient Experience Scale (HPES) domain scores and Short Form Survey (SF-36) Physical Functioning compared to placebo, with 93% achieving independence from conventional therapy [39,41]. It normalized 24-h urine calcium and was well-tolerated, with mild to moderate adverse events in 82% of treated participants and no study-related withdrawals [41].

An analysis evaluates palopegteriparatide’s impact on renal function in adults with chronic hypoparathyroidism through 104 weeks of the PaTHway trial, a phase III study featuring a 26-week randomized, double-blind, placebo-controlled period followed by a 156-week open-label extension (OLE) [42]. This post hoc analysis of the phase III PaTHway trial highlights palopegteriparatide’s sustained renal safety and suggests that PTH replacement therapy may preserve and even improve renal function in adults with chronic hypoparathyroidism while ensuring independence from conventional therapy [42]. In the PaTHway study, within 26 weeks the 600 mean 24-h urine calcium sustained levels below 6.2 mmol/day through week 104 (4.0 ± 2.3 mmol/day). No nephrolithiasis cases occurred, and most treatment-emergent adverse events (TEAEs) were mild or moderate, with no new safety concerns [42].

A study conducted by Clarke and colleagues revealed that by week 52, 81% (63 out of 78) of participants met the multicomponent efficacy endpoint, while 95% (74 out of 78) achieved independence from conventional therapy, with none requiring active vitamin D [43]. Patient-reported outcomes indicated sustained improvements in quality of life, physical functioning, and overall well-being. Bone mineral density Z-scores decreased toward age- and sex-matched norms from baseline to week 52. In addition, mean 24-h urine calcium excretion dropped from 376 (168) mg/day at baseline to 195 (114) mg/day at week 52. Most treatment-emergent adverse events were mild or moderate, and none resulted in trial discontinuation during the OLE [43]. In clinical trials, palopegteriparatide treatment allowed patients to achieve independence from conventional therapy (no active vitamin D and ≤600 mg/day elemental calcium) while maintaining serum biochemistries within normal ranges [42]. Safety was assessed through 24-h urine calcium excretion and TEAEs. At week 104, 93% (76/82) of participants remained in the trial. Among them, 82% had normal albumin-adjusted serum calcium levels (2.07–2.64 mmol/L), 97% were independent from conventional therapy, and none required active vitamin D [42].

Emerging treatments promise to revolutionize hypoparathyroidism (HypoPT) management. A long-acting PTH analog (eneboparatide) is in phase III trials, while a calcilytic (encaleret) is being tested for autosomal dominant hypocalcemia type 1 [39]. Eneboparatide, a long-acting PTH analog and PTH receptor 1 agonist, rapidly and sustainably lowered 24-h urinary calcium (uCa) levels, even in hypercalciuria patients. It slightly increased bone turnover markers while stabilizing bone mineral density (BMD), indicating a return to physiological bone turnover [44]. The treatment enabled independence from conventional therapy, maintained serum calcium within the target range, normalized uCa excretion, and supported balanced bone turnover restoration [44]. Encaleret corrected hypocalcemia and reduced hypercalciuria during inpatient and 24-week outpatient periods. It increased intact PTH, magnesium, and 1,25-dihydroxyvitamin D levels [45]. Bone turnover markers rose but remained normal in nine of thirteen participants. No serious adverse events were reported. Encaleret restored physiologic mineral homeostasis in 13 autosomal dominant hypocalcemia type 1 (ADH1) participants [45].

Palopegteriparatide is administered once daily via subcutaneous injection, has a long half-life of 60 h [36], and exhibits various adverse effects, including injection site reactions, vasodilatory signs and symptoms, headache, diarrhea, back pain, hypercalcemia, and oropharyngeal pain [38]. It was developed by Ascendis Pharma Bone Diseases A/S and approved by the FDA in 9 August 2024 [19].

### 3.2. Pegulicianine (Lumisight^TM^)

**Figure 5:**
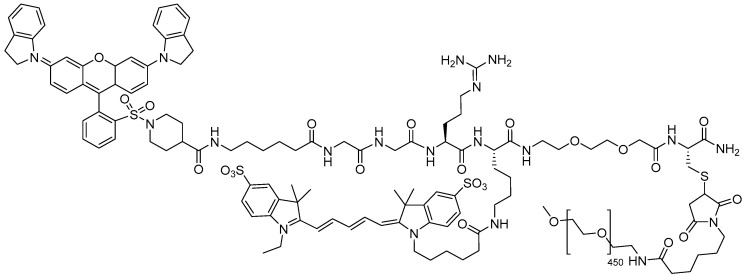
Chemical structure of pegulicianine (Lumisight^TM^).

Pegulicianine is an optical imaging agent featuring a GGRK linker and a branched side chain containing Cys within its structure (Figure 5). It is designed for fluorescence imaging in adults with breast cancer, used as an adjunct to detect cancerous tissue within the resection cavity intraoperatively after removing the primary specimen during lumpectomy [37]. Pegulicianine, used with the Lumicell Direct Visualization System (DVS) after lumpectomy, fluoresces in cancer cells, providing real-time guidance for surgeons to detect and remove otherwise undetectable cancerous tissues, enhancing surgical precision [46].

**Figure 6:**
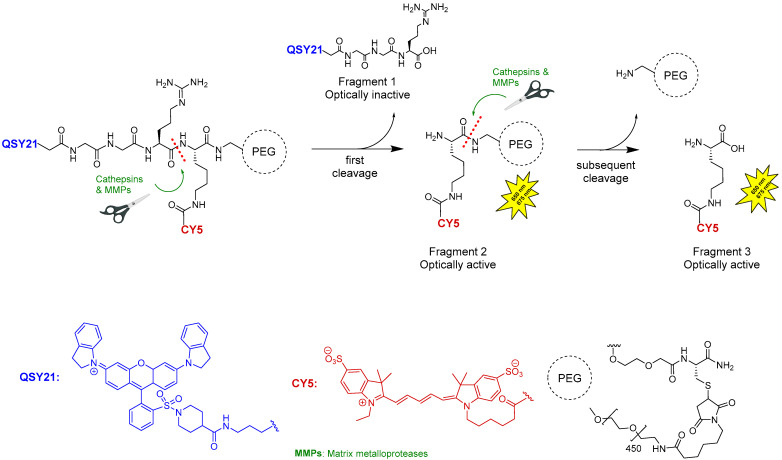
Fluorescence mechanism of pegulicianine.

Pegulicianine is a prodrug that remains optically inactive until cleaved by cathepsins and matrix metalloproteases (MMPs), enzymes found in higher concentrations around tumors and tumor-associated cells [37]. Cleavage generates “fragment 2” and “fragment 3”, which are fluorescent metabolites with peak absorption at 650 nm and emission at 675 nm, while “fragment 1, QSY21” contains a fluorescence quencher that keeps the intact molecule inactive (Figure 6) [37,47,48].

Pegulicianine is activated by enzymes within the tumor and stromal cells at its invasive front. Its fluorescence increases when the tumor is near the edge of the lumpectomy specimen, peaking when tumor is present at the inked margin [49]. This property helps guide surgeons to resect additional tissue in these areas, achieving appropriately wide negative margins. Converting positive margins after standard lumpectomy to adequate negative margins reduces the need for second surgeries, even when no tumor is found in the DVS-guided margins, underscoring the utility of this technology [49]. A study by Hwang and colleagues showed that fluorescence-guided surgery (pFGS) with guided shaves reduced the need for reexcision by 19% in patients undergoing breast-conserving surgery [50].

Lumpectomy offers survival rates comparable to mastectomy, but incomplete tumor removal increases recurrence and mortality risks [51]. Pathological margin evaluation, conducted post-surgery, may reveal invasive tumors or ductal carcinoma in situ (DCIS) within 2 mm of the margin, which often require additional surgery. Pegulicianine, as a fluorescent imaging agent, is injected pre-surgery and highlights residual tumors in real time during lumpectomy, guided by a handheld probe and tumor detection algorithm [37,51]. Studies confirm that pegulicianine pFGS effectively identifies residual tumors, reducing the need for reoperations [51]. This minimizes the need for additional surgery, reducing patient burden and associated healthcare costs [51]. Tissue concentrations of pegulicianine and its metabolites, including fluorescently labeled lysine, have shown that pegulicianine is selectively distributed to tumors, where it is activated by proteases [47].

The efficacy and safety of lumisight were assessed in a multicenter, intra-patient-controlled trial (NCT03686215) involving 357 breast cancer patients undergoing lumpectomy. Image-guided surgery with the lumicell DVS was performed post-standard lumpectomy, and tissue with a positive lumisight signal was resected using a cavity shave procedure [52,53]. While pegulicianine shows promise, its sensitivity (49.1%) and specificity (86.5%) indicate it is highly specific for cancer-free regions but moderately sensitive in detecting all cancerous areas [52,53]. It should complement, not replace, surgical judgment and imaging. Additionally, 43% of patients had false positives, risking unnecessary tissue removal, and 8% had false negatives, potentially retaining residual disease [52]. In cancer diagnosis, false positives cause unnecessary anxiety, invasive procedures, and life disruptions, while false negatives delay diagnosis, allowing cancer to progress, potentially worsening treatment outcomes and survival rates.

Pegulicianine is administered intravenously and showed hypersensitivity including anaphylaxis as adverse effects [37]. Patients should be evaluated for a history of contrast media hypersensitivity, and emergency resuscitation drugs, equipment, and trained personnel must always be readily available [37]. Moreover, chromaturia has been observed, suggesting that metabolites may be excreted through the kidneys [37]. Of 230 trial participants, one patient with a history of contrast agent allergy experienced an anaphylactic reaction but recovered fully [50]. Per-margin analysis showed pegulicianine fluorescence-guided surgery (pFGS) significantly improved sensitivity compared to standard pathology. Pegulicianine demonstrated a safety profile consistent with other imaging agents used in breast-conserving surgery (BCS) and reduced the need for second surgeries through pFGS-guided intraoperative excisions [50]. It was developed collaboratively by Duke University, Lumicell, and the National Cancer Institute (USA) [47] and received FDA approval with Lumicell as the originator on 17 April 2024 [20].

### 3.3. Levacetylleucine (Aqneursa^TM^)

**Figure 7:**
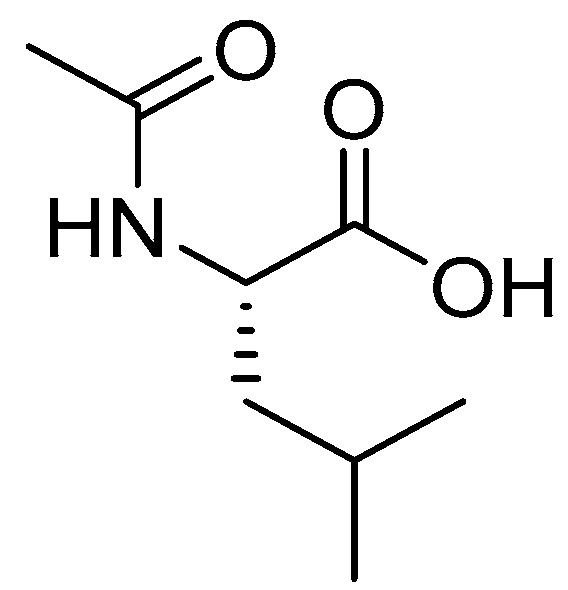
Chemical structure of levacetylleucine (Aqneursa^TM^).

Levacetylleucine is an acetylated Leu which is used for treating neurological manifestations of Niemann–Pick disease type C (NPC) in adults and pediatric patients weighing ≥15 kg (Figure 7) [54]. Although levacetylleucine (Aqneursa™) is not a peptide, we chose to include it to emphasize the significance of amino acids as the fundamental building blocks of peptides. NPC is a rare genetic disorder caused by mutations in the NPC1 (95%) or NPC2 (5%) genes, disrupting cholesterol trafficking and leading to organ damage and progressive neurological symptoms [55]. Symptoms range from fatal infantile regression to later-onset forms with splenomegaly, gaze palsy, ataxia, dysarthria, and dementia. The average life expectancy is around 13 years [56,57]. Currently, there is no cure for NPC. The ganglioside synthesis inhibitor miglustat is the first approved treatment in many countries, showing potential to slow disease progression [58]. Recent clinical trials have demonstrated that the modified amino acid *N*-acetyl-l-leucine (NALL) can improve clinical signs and symptoms in NPC patients [59].

Although the mechanism of action of *N*-acetyl-dl-leucine remains unclear, studies have investigated its effects. In models of Sandhoff’s disease, which is a rare neurodegenerative disorder marked by GM2 ganglioside accumulation in lysosomes, levacetylleucine normalized glucose and glutamate metabolism, enhanced autophagy, and increased levels of superoxide dismutase, a reactive oxygen species scavenger [60]. In Niemann–Pick type C models, levacetylleucine improved mitochondrial energy metabolism and altered glucose and antioxidant pathways [61]. Notably, despite l-leucine’s role as a mammalian target of rapamycin (mTOR) activator, no changes in mTOR levels or phosphorylation were observed after treatment with *N*-acetyl-dl-leucine or its enantiomers in these models [60,61].

A total of 60 patients aged 5 to 67 years were enrolled. In NPC patients, 12 weeks of NALL treatment improved neurologic status compared to placebo. Secondary endpoints generally supported these findings, though they were not adjusted for multiple comparisons. No adverse events relating to NALL or placebo were reported. One death, due to aspiration pneumonia after a planned gastrostomy tube placement, was not treatment related. Plasma, urine, vital signs, and electrocardiogram (ECG) results were normal or clinically insignificant [62].

Levacetylleucine is administered orally and has the following adverse effects with an incidence of ≥5%: abdominal pain, dysphagia, upper respiratory tract infections, and vomiting [54]. It was developed by IntraBio Inc and approved by the FDA on 24 September 2024 [21].

## 4. Conclusions

Peptides and oligonucleotides are emerging as a preferred treatment for challenging diseases, particularly genetic disorders. In 2023, a peptide-based drug became the first approved treatment for Rett Syndrome, marking a significant milestone [24]. Similarly, this year, NPC disease was treated with a simple acetylated amino acid, highlighting the critical role of peptides and their building blocks in saving lives. Peptides provide an effective therapy for hypoparathyroidism, with PTH replacement offering a superior physiologic treatment compared to conventional therapy with active vitamin D and calcium, which address hypocalcemia but fail to restore normal PTH function.

The recent approvals of oligonucleotide therapies, imetelstat and olezarsen, further highlight the progress in oligonucleotide-based approaches. These therapies showcase advancements in targeted delivery systems and therapeutic precision, providing novel solutions for unmet medical needs in hematologic disorders and rare genetic diseases. Continued innovation in chemical design and delivery platforms is anticipated to broaden the therapeutic applications of both peptides and oligonucleotides, driving improvements in efficacy, safety, and patient outcomes.

The decrease in the number of approved TIDES in 2024 can be attributed to pharmaceutical companies focusing on scaling up the production of existing pharmaceuticals, a challenging endeavor [63,64]. This shift in focus is compounded by a global shortage of critical molecules, such as glucagon-like peptide-1 (GLP-1), which has demonstrated multifunctional roles in treating diabetes, obesity, and, more recently, Alzheimer’s disease in addition to providing cardiovascular benefits [65,66]. However, this does not imply a decline in the importance of TIDES. On the contrary, tracing the number of TIDES in development pipelines reveals significant progress in the field. For example, Bicycle Therapeutics has ten peptides in development [67], Alnylam is advancing seventeen oligonucleotides [68], Zealand Pharma is working on six peptides [69], Novo Nordisk has nine peptides and four oligonucleotides [70], and Eli Lilly and Company is progressing with six peptides and four oligonucleotides across various clinical phases [71]. The peptide market, valued at USD 10 billion in 2023, is expected to grow substantially to USD 106 billion by 2033, with a compound annual growth rate (CAGR) of 10.8% [72].

AI and machine learning accelerate peptide discovery through rapid data analysis, candidate identification, and predictive modeling. The rise of AlphaFold and AI further advances peptide-based drug discovery; interested readers are referred to relevant studies for further insights [73,74,75,76,77].

## Figures and Tables

**Table 1:** Summary of FDA-approved TIDES in 2024.

No.	Active Ingredient (Trade Name)	Indication	Therapeutic Target	Administration Route	Ref.
Oligonucleotides					
1	Imetelstat (Rytelo^TM^)	Treatment of adult patients with low- to intermediate-1 risk myelodysplastic syndromes (MDSs) with transfusion-dependent anemia for whom erythropoiesis-stimulating agents (ESA) are not suitable.	Telomerase RNA-template	Intravenously	[17]
2	Olezarsen (Tryngolza^TM^)	An adjunct to diet to reduce triglycerides in adults with familial chylomicronemia syndrome (FCS).	APOC-III mRNA	Subcutaneously	[18]
Peptides					
3	Palopegteriparatide (Yorvipath^TM^)	To treat hypoparathyroidism in adults.	PTH1R	Subcutaneously	[19]
4	Pegulicianine (Lumisight^TM^)	Adjunct to detect cancerous tissue within the resection cavity intraoperatively after removing the primary specimen during lumpectomy.	Cancerous tissue within the resection cavity	Intravenously	[20]
5	Levacetylleucine (Aqneursa^TM^)	To treat Niemann–Pick disease type C (NPC).	Unknown	Oral suspension	[21]

APOC, apolipoprotein C-III; FCS, familial chylomicronemia syndrome; MDSs, myelodysplastic syndromes; PTH1R, parathyroid hormone analog.

## Data Availability

Data sharing is not applicable.
